# Influence of gestational salt restriction in fetal growth and in development of diseases in adulthood

**DOI:** 10.1186/s12929-016-0233-8

**Published:** 2016-01-20

**Authors:** Hiroe Sakuyama, Minami Katoh, Honoka Wakabayashi, Anthony Zulli, Peter Kruzliak, Yoshio Uehara

**Affiliations:** 1 Division of Clinical Nutrition, Faculty of Home Economics, Kyoritsu Women’s University, 2-2-1 Hitotsubashi, Chiyoda, Tokyo 101-8437 Japan; 2 The Centre for Chronic Disease Prevention & Management (CCDPM), Western CHRE, Victoria University, St Albans, Australia; 3 2nd Department of Internal Medicine, Faculty of Medicine, Masaryk University, Pekarska 53, 656 91 Brno, Czech Republic; 4 Laboratory of Structural Biology and Proteomics, Central Laboratories, Faculty of Pharmacy, University of Veterinary and Pharmaceutical Sciences, Brno, Czech Republic

**Keywords:** Salt restriction, Low birth weight, Low birth rate, Programing, Growth retardation, Hypertension, Salt sensitivity, Insulin resistance, Dyslipidemia

## Abstract

Recent studies reported the critical role of the intrauterine environment of a fetus in growth or the development of disease in adulthood. In this article we discussed the implications of salt restriction in growth of a fetus and the development of growth-related disease in adulthood. Salt restriction causes retardation of fatal growth or intrauterine death thereby leading to low birth weight or decreased birth rate. Such retardation of growth along with the upregulation of the renin angiotensin system due to salt restriction results in the underdevelopment of cardiovascular organs or decreases the number of the nephron in the kidney and is responsible for onset of hypertension in adulthood. In addition, gestational salt restriction is associated with salt craving after weaning. Moreover, salt restriction is associated with a decrease in insulin sensitivity. A series of alterations in metabolism due to salt restriction are probably mediated by the upregulation of the renin angiotensin system and an epigenetic mechanism including proinflammatory substances or histone methylation. Part of the metabolic disease in adulthood may be programmed through such epigenetic changes. The modification of gene in a fetus may be switched on through environment factors or life style after birth. The benefits of salt restriction have been assumed thus far; however, more precise investigation is required of its influence on the health of fetuses and the onset of various diseases in adulthood.

## Background

Salt is an essential nutrient for all cells and a reduction in salt intake results in the collapse of cardiovascular circulation. Humans possess more salt-retaining systems, e.g., hemodynamic, hormonal and renin angiotensin systems than salt-excreting mechanisms. Therefore, an imbalance between salt intake and salt-retaining systems is believed to be responsible for the onset of cardiovascular disease in adulthood.

With regard to the intrauterine environment, however, the effects of salt intake by mothers on the fetal growth have not yet been addressed. In fact, the fetal mechanisms for coping with a low-salt environment or the impact of excessive salt restriction on the fetal growth and health have not been investigated. Given their intrauterine status, fetuses are more subject than adults to a low-salt environment and are influenced by salt restriction-related hemodynamic and hormonal alterations in their mothers as well as by their own homeostatic changes.

Recent studies have found an association between intrauterine growth retardation and cardiovascular dysfunction with structural changes in both animals and humans. In this sense, it would be intriguing to determine the pathophysiological role of maternal salt metabolism in the fetal growth and to examine whether growth retardation due to excessive salt restriction is associated with diseases in adulthood.

Accordingly, this review focuses on recent studies concerning the effects of excessive salt restriction on fetal growth and reviews the possible mechanism underling this risk. It is also within our scope to discuss the association of growth retardation due to salt restriction with hypertension and metabolic diseases in adulthood, with special reference to epigenetic and gene-related mechanisms.

### Salt restriction and excessive salt intake

In the field of hypertension research, a salt intake of 6 g per day (equivalent to 2.4 g sodium) has long been recommended for hypertensive patients, regardless of the presence of or absence of diabetes or chronic kidney diseases [1]. However, the average salt intake worldwide is estimated to be approximately 9.87 (5.45–13.77) g per day according to public information [2]. The average salt intake in the United State is approximately 9 g per day, and less than 5 % US of the population consumes less than 3.75 g per day [3]. In Japan, the estimated salt intake has remained at approximately 10–15 g per day for decades [4]. Salt intake depends on age, gender and ethnicity, and this likely explains the lack of consensus regarding defined amounts of sat indicative of restriction or excessive intake. In general, however, the target amount for salt restriction is less than 6 g per day, and 6–12 g per day is considered moderate or normal salt intake. A salt intake exceeding 12–15 g day might be considered a high-salt intake in clinical settings. Dietary Guidelines for Americans 2010 recommends a maximum dietary sodium intake of 2300 mg per day for the general population, and the World Health Organization recommends 2000 mg of sodium; however, most Americans consume a higher level of sodium, according to data from the National Health and Nutrition Examination Survey [4]. In Canada, a daily salt intake of 3.75 g salt per day is recommended; however, an intake of less than 3 g per day has been unattainable thus far on a public population. Small clinical studies, however, have studied severe low-salt groups, defined as those with a salt intake of less than 1 g per day. Thus, the definition of salt restriction likely varies among studies, and in the present review we have attempted to specify the amount of salt whenever possible.

The findings from animal studies are much clear. The salt content in regular chow used for rodent colony maintenance is approximately 0.75 % (w/w). In the field of hypertension research, a low-salt diet contains 0.3 % salt (w/w) and a no salt diet contains less than 0.1 % salt (w/w) [5, 6]. In other words, animals fed a low-salt diet consume less than half of the usual salt intake. In studies using Dahl salt sensitive (Dahl S) rats, a high-salt diet is defined as containing 4 or 8 % salt (w/w), according to the definition given by the American Heart Association hypertension council [5, 6]. A Dahl S rat weighing 300 g usually consumes approximately 30 g chow per day, or approximately 0.3 g salt /kg BW per day for a 0.3 % low-salt diet and 8 g salt/kg BW per day for an 8 % high-salt diet.

Moreover, there are notably no standard methods with which to assess salt intake in clinical settings. In small clinical studies, salt intake is accurately assessed by determining of the salt content of foods; however, in large population-based epidemiological studies, it is impossible to obtain 24 h urine samples to determine the urinary excretion of sodium as an index of salt intake. Thus, salt intake is usually estimated from interviews concerning ingested foods or via spot urine collection, which might result in variability in the reported amounts of salt intake in the literature [7, 8]. Accordingly, in this review, we have provided information on the methods used to determine salt intake to the extent possible.

### Salt restriction and health risk in offspring

For more than 50 years, the role of salt in health has been an important topic in the field of public health as well as in cardiovascular research. Increased salt intake has been implicated in hypertension and various other vascular diseases. Whether a lower salt intake is better for health from a public health standpoint has remained a matter of debate worldwide [9, 10]. To our surprise, however, a recent meta-analysis of 25 previous studies on salt intake and an average 3.7-y observational study of 101,945 people in 17 countries wherein salt intake was assessed by determining urinary sodium excretion demonstrated an association between low-salt intake and an increase in cardiovascular events/death, compared with normal salt intake [10, 11]. Graudal et al. reported that low sodium intake (less than 115 mol per day, equivalent to 6.67 g salt) and high sodium intake (more than 215 mol per day, equivalent to12.47 g salt ), relative to the usual sodium intake (115–215 mol per day, equivalent to 6.67–12.47 g salt), were both associated with increased mortality. There was no difference between low usual sodium intake (115–165 mol per day, equivalent to 6.67–9.57 g salt) and high usual sodium intake (165–215 mol per day, equivalent to 9.57–12.47 g salt). This is consistent with a J-shaped association between sodium intake and health outcomes [10]. Similarly, a daily sodium intake of 4–5.99 g, equivalent to 10–15 g salt, estimated on the basis of measured urinary excretion, was associated with a lower risk of death and cardiovascular events than either a higher (≥7.0 g sodium per day, 17.5 g salt) or lower salt intake (<3 g sodium per day, 7.5 g salt) [11]. These studies estimated an optimal salt intake of approximately 8-10 g salt per day. The mechanism by which low-salt intake confers this risk is unclear. However, it is noted that the benefits of blood pressure changes appear to be small because the blood pressure reduction due to salt restriction is slight in the range of moderate salt intake (7.5–12.5 g salt per day) [12]. On the other hand, renin angiotensin system is enhanced under conditions of salt restriction (<3.0 g sodium per day, 7.5 g salt) [11], and this presumably promotes cardiovascular and renal injury mechanisms independently of blood pressure. Despite such well-designed studies on salt and cardiovascular diseases in adulthood, few studies have addressed the role of gestational salt intake in the health of mothers and their neonates. Intriguingly, however, some studies suggest that a slight change in intrauterine circumstances influence fetal growth. In particular, intrauterine growth retardation (IUGR) due to salt restriction might be possibly related to the underdevelopment of cardiovascular or metabolic systems. An interesting hypothesis has been proposed that some forms of cardiovascular or metabolic diseases in adulthood are programmed during fetal growth in the mother’s uterus.

### Impaired growth and gestational salt restriction

Salt restriction is associated with low birth and survival rates and low birth weight. In an animal study, intake of low-salt (0.3 % NaCl, w/w) chow during mating and gestation is associated with low birth and survival rates of pups of Dahl salt-sensitive rat pups, compared with pups from mothers fed a high-salt (4 % NaCl, w/w) diet (Table 1) [13]. Low-salt intake during mating and pregnancy causes an approximately 50 % decrease in birth rate, with a significantly lowered birth weight. These are the results of spontaneous abortion or impaired fetal growth due to salt restriction during pregnancy. The low-salt chow utilized is approximately 50 % less salt than the regular chow (0.75 % NaCl, w/w) for maintaining a colony [13, 14]. This suggests that moderate salt restriction during the perinatal period could be a risk to the normal intrauterine growth of a fetus.

**Table 1:** Number of mothers that delivered pups

Group	Diet (NaCl, w/w)		Mother (No)			Statistics^a^		
	Mating	Pregnant	Total	Delivered**	Babies/Mother^b^	χ^2^	*p*	vs
MHPR	4.0 %	0.75 %	7	7	7.4 ± 2.3			
MLPR	0.3 %	0.75 %	7	4	8.7 ± 1.7	3.82	0.050	MHPR
MRPR	0.75 %	0.75 %	8	7	8.2 ± 1.4	0.94	0.332	MHPR
						1.76	0.184	MLPR
						1.45	0.228	MRPH
						2.33	0.127	MRPL
MRPH	0.75 %	4.0 %	11	11	10.2 ± 1.3			
MRPL	0.75 %	0.3 %	11	6	8.8 ± 3.0	6.87	0.011	MRPH

MHPR, mothers fed the high-salt (4 % NaCl, w/w) diet during mating period and the regular (0.75 % NaCl, w/w) chow during pregnant period; MLPR, mothers fed the low-salt (0.3 % NaCl, w/w) diet during mating period and the regular chow during pregnant period; MRPR, mothers fed the regular chow during mating period and during pregnant period; MRPH, mothers fed the regular chow during mating period and the high-salt diet pregnant period; MRPL, mothers fed the regular chow during mating period and the low-salt diet during pregnant period. Total, the total number of mothers; delivered, the number of mothers that gave birth to pups; non-delivered, the number of mothers that failed to give birth to pups. **The differences in delivered rats were assessed by χ^2^ test (df = 1). 0.75 % NaCl (w/w) chow (regular chow) is usually used for maintaining of rat colonies. ^b^mothers that delivered babies.

Cited from Chou R et al. Journal of Nutrition and Metabolism volume 2014. Article ID 212089, (ref# [13]).

**Fig. 1:**
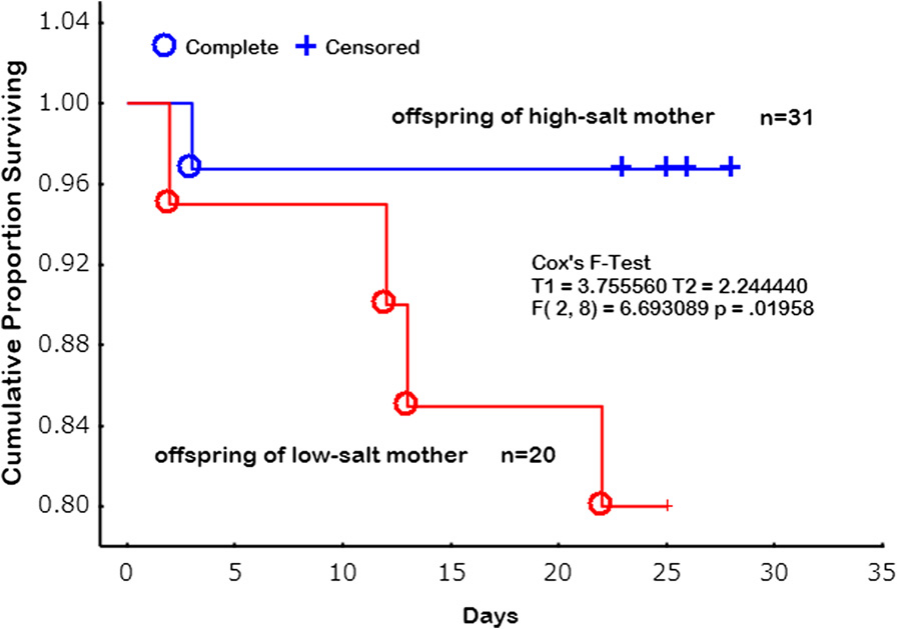
Cumulative proportion of offspring survival (Kaplan Meier analysis). Survival prognosis was evaluated from their birth of the rat pups to weaning. Open circles refer to the death (complete) of pups from high-salt (4%NaCl, w/w) intake mothers; solid circles refer to the (complete) of pups from a low-salt (0.3%NaCl, w/w) intake mothers. plus (+), censor. The difference was analyzed by Cox's F-Test (T1 = 3.755, T2 = 2.244, F(2, 8) = 6.6930, *p* = 0.01958). Cited from Chou R et al. (2014) Journal of Nutrition and Metabolism volume 2014. Article ID 212089, (ref# [13]).

Moreover, the pup survival rate during lactation was 95 % for mothers on a high-salt diet during pregnancy, whereas it was as low as 64 % for mothers on the low-salt (0.3 % NaCl, w/w) diet (Fig. 1) [13]. It is not clear whether this is related to behavior changes in mothers due to salt restriction or to babies that possibly have defects critical in intrauterine environments. As shown in Kaplan Meier analysis there are two types of death, i.e., death just after birth and that during normal growth of pups while taking milk. Since the death is associated with cannibalism, it is noted that the salt restriction could cause behavior changes in mothers. More precise investigation is needed. Moreover, de Siqueira et al. also reported that Wistar rats with a low-salt intake (0.15 % NaCl, w/w) in the second half of gestation had low birth weight and size, thereby suggesting that the salt restriction (0.15 % NaCl, w/w) more likely affects the intrauterine development of the organs of fetuses than normal sodium diet (1.3 % NaCl, w/w) [15].

**Fig. 2:**
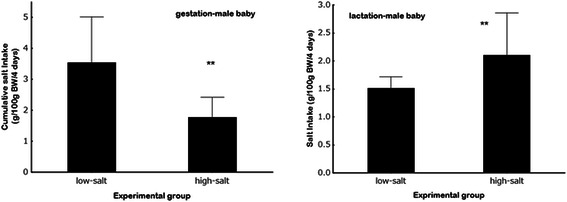
Amounts of saline solutions in the male baby rats from low-and high-salt mothers during gestation and lactation. Cumulative salt intake for the 4-day in male baby rats from low- (0.3 % NaCl, w/w) and high-salt (4%NaCl) mothers during gestation (left graph) and lactation period (right graph) **p < 0.005 vs the respective values of low-salt (0.3 % NaCl, w/w) mothers. Cited from Hara A et al. (2014) Food and Nutrition Sciences Vol.5 No.19, PP. 1904–1913 (ref# [23]).

In humans, the direct evidence of the etiological association of gestational salt restriction and impaired growth of neonates is limited. Recently, however, there have been a growing number of studies supporting the hypothesis. Originally, it was reported that severe morning sickness and hyperemesis gravida is often associated with low birth weight or premature labor and the loss of salt resulting from vomiting is believed to be responsible for both the low birth rate and low birth weight [16–18]. Moreover, Shirazki et al. found that babies with low blood sodium concentrations resulting from their mother’s low-salt intakes during pregnancy are more likely to be underweight at birth [19]. Salt restriction or mineralofluid loss due to vomiting in pregnancy influences physical status, and babies born of mothers with extracellular volume loss by polyethylene glycol more likely crave salt after birth [19, 20]. Low-salt (0.3 % NaCl, w/w) solely during gestation is related to the increase of salt appetite in pups, but after birth the relation disappears. Low-salt diet (0.3 % NaCl, w/w) in pregnancy caused craving for salt in pups compared with high-salt (4 % NaCl, w/w) mothers whereas after weaning, salt appetite increases in babies fed a high-salt (4 % NaCl, w/w) diet compared with those fed a low-salt (0.3 % NaCl, w/w) diet (Fig. 2) [21–23]. It is also known that in premature babies, physical and mental abilities are improved in children whose diets are supplemented with salt [24].

### Mechanism of impaired growth due to salt restriction

The reasons for fewer or low-birth-weight pups being born of pregnant mothers with salt restrictions remain unclear. However, low birth weight in response to salt-restriction during pregnancy is due to alterations of uterine-placental perfusion [25]. It has also been reported that salt is critical for the development of the glial (immune) cells in the brain. In fact, it is reported that salt restriction of 0.022–0.04 % sodium diet caused retardation of brain development in association with less consumption of diet, low weight and low survival rate during lactation in Sprague Dawley rats [26]. Infants with low sodium intake may experience poor neurological function in early adolescence, and low-salt intake impairs the normal development of the nerve sheath in a fetus. Growth inhibition of astrocytes in the neonate brain, which occurs because of low-salt intake during pregnancy, is possibly involved in the dysfunction of the autonomic system in adulthood, thereby affecting hemodynamic or metabolic changes in adulthood. Although this hypothesis is interesting, further clinical trials are required for validation.

Decreased renal blood flow due to low-salt intake usually enhances the activity of the renin angiotensin system, and the enhancement conceivably affects the intrauterine growth of infants of mothers with salt restrictions. In fact, the overexpression of the renin angiotensin system in transgenic mice causes a placenta-fetus imbalance and leads to pre-eclampsia [27–29]. The increase in angiotensin II due to salt restrictions is assumed to cause a placenta-fetus imbalance, and if so, it may result in low birth rate or low birth weight. Moreover, recent studies have reported an angiotensin II-mediated epigenetic mechanism for disease expression or impaired kidney and organ growth during pregnancy [30–33]. Events that are triggered after a low-salt challenge, i.e., enhanced regulation of rein angiotensin system, are presumed to be involved in gene modification that become overt through influences after birth. Less than 3 g sodium/day is expected in general to trigger renin-angiotensin axis. Moreover, it is well reported that during critical developmental periods, salt restriction might affect fetal hormonal, vascular, and renal systems to regulate fluid homeostasis in the fetus [34]. Both the neurohypophysial hormones arginine vasopressin and oxytocin participating in volume regulation in human pregnancy and modulating vascular tone are reported to be impaired in severe sodium (0.46 g/day) restriction [35]. Such hormonal response might contribute in part to the impaired growth or death in pups from mothers on low-salt diet. The details are to be discussed later.

### Gestational salt intake and adult hypertension

Since Barker and investigators from other laboratories reported the association of birth weight and death due to ischemic heart disease [36], there has been a considerable amount of evidence in animal studies suggesting that low birth weight determines the onset of hypertension, insulin resistance and lipid dysfunction [37, 38]. The relationship has been consistently confirmed in humans as well [39–41]. Very large longitudinal study of 13,517 subjects born in Helsinki University Hospital between 1924 and 1944 clearly demonstrated that small size at birth and during infancy with accelerated weight gain from age 3 to 11 years predicts higher incidence of coronary heart diseases, type 2 diabetes and hypertension [42]. These diseases are integral components of metabolic syndrome, and it suggests that intrauterine process in such a low birth weight fetus programs the clustering of these disorders in adulthood.

Animal studies have provided some information on the mechanism that underlies the association. Recently, Benz and Amann reviewed the role of nephron development in the association of maternal nutrition and hypertension [43]. In humans, nephrogenesis occurs from weeks 5 to 36 of gestation, with the most critical period being the mid-second trimester until 36 weeks; fetuses are very sensitive to genetic and environmental factors such as maternal diet during this 16-week period. This critical period is in accordance with the data from de Siqueira et al. suggesting low-salt intake in the second half of gestation is associated with low birth weight and size [15]. Such slight changes in intrauterine circumstance that cause low birth weight hamper normal kidney development [44, 45]. A decreased number of nephrons could conceivably cause salt sensitivity and thereby developing hypertension in adult offspring. Indeed, Simonetti et al. found that children with growth-restriction have a risk for reduced renal mass as determined by ultrasound and increased salt sensitivity [46]. A decreased number of nephrons during gestational development or by acquired kidney diseases reduce the ability of nephrons to handle sodium excretion, which leads to the development of hypertension.

Moreover, salt-restriction during pregnancy enhances salt-craving in Dahl pups after weaning [13]. Salt-restriction (0.3 % NaCl, w/w) in gestation causes an increase in salt-appetite, but the taste of salt does not change. The association of salt-restriction with an increase in salt appetite is observed in both male and female pups. In contrast, high-salt (4 % NaCl, w/w) intake in mothers during lactation increases the salt appetite in the pups after weaning. Salt loss in pregnant rats resulting from polyethylene glycol intake which produces extracellular dehydration increases salt-appetite [21]. In humans it has been well documented that morning sickness and salt-loss due to hormonal dysfunction in neonates in turn enhances salt-appetite later in their babies [16–20]. Interestingly, Shirazki et al. clearly demonstrated that in low birth weight children, salt appetite in children aged 10 years old is negatively related to the neonatal serum sodium [20]. In general, in animals and in humans thirst and sodium appetite are programmed by the developmental environment [47]. Increased salt-appetite and enhanced salt-sensitivity due to salt-restriction concurrently increase blood pressure [46]. It is well known that baby Dahl salt-sensitive pups are more sensitive to salt-loading than adult rats.

Salt-restriction decreases renal blood flow and triggers the secretion of renin from the kidney. The increased plasma renin concentrations initiate the breakdown of renin substrate, angiotensinogen, and finally, produce the octapeptide, angiotensin II. The upregulation of angiotensin II probably influences food preferences. In very young, normal female subjects, the consumption of total lipids, cholesterol, and unsaturated free fatty acids is higher in those with the MM/MT genotype of AGTMet235 [48]. In this context, some studies have been performed to postulate the role of intracerebral angiotensin II in water and salt drinking, and it is found that angiotensin II infusion in the cerebral fluid enhances behavior of drinking water and salt-appetite [49–51]. Salt-restriction evokes activity of the renin angiotensin system, and its upregulation due to salt-restriction during fetal growth might be associated with an increased salt-appetite. More directly, sodium depletion produced marked increases in the dam's plasma angiotensin II and aldosterone concentrations. During prenatal sodium depletion, the activation of the renin angiotensin system rather than the loss of sodium itself is responsible for the modified salt intake behavior [52].

Such changes in renin angiotensin system increases blood pressure through vasoconstriction, a decrease in sodium excretion in the kidney, enhanced adrenergic nervous system and remodeling of the cardiovascular structure [22]. There is a great deal of evidence suggesting that intrauterine growth retardation (IUGR) or preeclampsia is associated with an increase in angiotensin II and that renin angiotensin system plays a critical role in under-development of intrauterine growth [27, 53–57]. However, the relationship of renin angiotensin system and organ growth is somewhat controversial. An intact renin angiotensin system is necessary for kidney function, and genetic defects of renin angiotensin system are associated with kidney abnormalities [58–61]. More directly, suppression of renin angiotensin system by losartan shortly after birth is associated with a significantly lower nephron number with hypertension [62, 63]. Taken together, alterations of the intrauterine environment might be associated with either suppression or overexpression of components of the renin angiotensin system which then potentially contribute to hypertension by interfering with nephrogenesis.

### Salt restriction and insulin resistance

An increasing amount of evidence has suggested that the programming occurring during gestation may play an important role in the development of metabolism-related disorders during adulthood in humans [64–66]. Low birth weight resulting from salt restriction during pregnancy influences insulin sensitivity and dyslipidemia. Animal studies have demonstrated that lower glucose uptake and higher plasma cholesterol and triacylglycerol concentrations were observed in offspring of salt-restricted (0.15 % NaCl, w/w) Wistar rat dams than those in offspring from mothers fed a normal-salt (1.5 % NaCl, w/w) diet [38]. Vidonho et al. reported that salt restriction (0.15 % NaCl, w/w) during pregnancy and lactation in Wistar rat dams decreases glucose uptake and insulin sensitivity in offspring determined by the glucose clamp method than high (4 or 8 % NaCl, w/w) or normal (1.3 % NaCl, w/w) salt diet [37]. This was accompanied by higher plasma cholesterol and triacylglycerol concentrations. However, blood pressure was not necessarily increased in offspring of low-salt (0.15 % NaCl, w/w) dams, which may be related in part to a different salt appetite in response to salt restriction (0.3 % NaCl, w/w) during pregnancy and lactation periods as demonstrated in our previous study [13]. The sensitivity to salt intake may differ according to growth stage, i.e., intrauterine, lactation and after weaning. Unfortunately, there have been few studies thus far that discuss the effects of salt in the respective life stage. In this sense, we will have to wait to determine the exact relationship between salt intake and effects in adulthood. Despite such limitations, it has been noted that salt restriction in pregnant mothers or chronic salt restriction in both animals and humans is associated with decreased glucose uptake and insulin sensitivity.

### Renin angiotensin system and insulin sensitivity

The association of salt restriction to a decrease in insulin sensitivity is explained to some extent by the enhancement of renin angiotensin system following chronic salt restriction. It has been well established in hypertension research field that enhanced activity of renin angiotensin system facilitates adrenergic nervous activity in animals and humans [22, 67]. Upregulation of the α-adrenergic nervous system increases sodium reabsorption in renal tubules and induces vasoconstriction, both of which increase blood pressure [68, 69]. Vasoconstriction by α-stimulation reduces blood flow in the muscles and β_3_ adrenoceptor stimulation increases lipolysis and gluconeogenesis, thereby mediating type-2 diabetes [70–72]. In this context, Ruivo et al. demonstrated using rats that α- and β-adrenoceptor blockades or L-arginine, improves glucose uptake as determined by the glucose clamp method, thereby increasing insulin sensitivity [73]. Low birth weight children show an increased heart rate with enhanced catecholamines and associated with sympathoadrenal overactivity after birth [74]. Because there are a bunch of studies suggesting that renin angiotensin system underlies the onset of the metabolic syndrome in adulthood, it is quite interesting that renin angiotensin system mediates the association of salt restriction during pregnancy with a decrease in insulin sensitivity in adulthood [75–77]. However, it is not clear whether renin angiotensin system is enhanced in intrauterine fetuses in salt-restricted mothers or if angiotensin II from mothers affects fetal growth through placental blood flow.

### Mechanism of insulin resistance by salt restriction

Renin angiotensin system evokes proinflammatory cytokines, i.e. tumor necrotizing factor (TNF)-α and enhances oxygen stress through NADPH oxidase activation [78–83]. The proinflammatory cytokines, i.e. TNF-α, interleukin (IL)-1β, IL-6 and resistin, and nitric oxide (NO) all act on adipocytes to induce an insulin-resistant state [78]. The alterations of subcellular signal transduction in insulin resistance have been extensively investigated. Salt restriction conceivably induces proinflammatory cytokines-mediated insulin resistance through upregulated renin angiotensin system; however, few articles thus far discussed subcellular signal transduction due to salt restriction. A recent study has found that high-salt (3.12 % sodium, w/w, equivalent to 7.8 % salt) diet increases insulin sensitivity in Wistar rats with increased GLUT4 gene expression, and increased insulin signaling, whereas low-salt (0.06 % sodium, w/w, equivalent to 0.15%salt) diet decreases insulin sensitivity with impaired insulin signaling [84]. Moreover, Prada et al. reported that the association of severe salt restriction (0.15 % NaCl vs high-salt 1.25 % NaCl diet) and insulin resistance and obesity is attributable to c-jun N-terminal kinase activity [85]. The kinase is involved in the pathophysiology of obesity and induces insulin resistance by increasing inhibitory IRS-1^ser307^ phosphorylation. This demonstrated that salt restriction increases c-jun N-terminal kinase activity and IRS-1 ^ser307^ phosphorylation, thereby increasing body weight, visceral adiposity, and blood glucose and insulin levels with insulin resistance.

### Epigenetics and salt restriction

Epigenetics, the variations in DNA methylation patterns and chromatin remodeling, provide an intriguing explanation for how environmental factors or intrauterine nutrition modify the risk for developing metabolic diseases in adulthood. However, there are limited data on the influence of early nutrition on epigenetics modifications [86]. One example of an epigenetic mechanism is that uteroplacental insufficiency-induced intrauterine growth restriction (IUGR), which predisposes to adult-onset insulin resistance, decreases postnatal insulin-like growth factor-1 (IGF-1) mRNA variants and the gene elongation mark histone 3 trimethylation of lysine 36 of the IGF-1 gene (H3Me3K36). H3M e3K36 is sensitive to the prenatal environment’s glucose level with resultant alteration of IGF-1 mRNA expression and ultimately vulnerability to adult-onset insulin resistance [87–89].

The epigenetic mechanism is very promising for explaining the role of salt restriction in developing insulin resistance in adulthood. It has not been proven whether the enhanced activity of renin angiotensin system due to salt restriction mediates indeed the epigenetic mechanism. Moreover, if epigenetic DNA modifications occur, there have been few studies thus far on phenotype expression resulting from the epigenetic changes. Renin angiotensin system enhances proinflammatory cytokines or oxygen stress and nFkB-related transcriptional process [81, 83, 90–92]. In this sense it seems quite interesting to define whether angiotensin II participates in epigenetic mechanism with alterations of oxygen stress. Taken together, the epigenetic hypothesis promises to explain the late onset of diseases in adulthood. However, the studies are in their infancy, and the implications of epigenetic involvement in phenotypic alterations in adulthood due to salt restriction in pregnant mothers remain to be elucidated.

## Conclusions

**Fig. 3:**
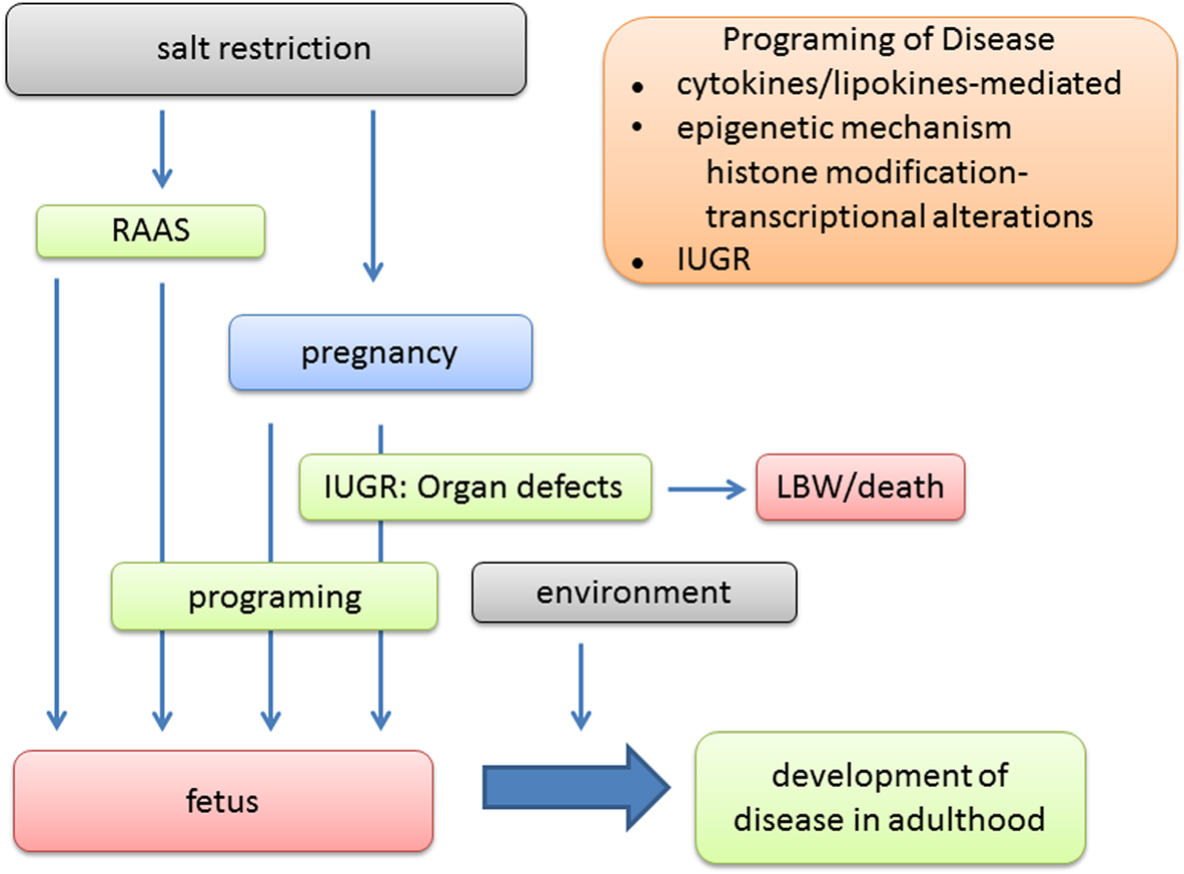
Prospective mechanism for development of disease in adulthood. RAAS, renin angiotensin aldosterone system; IUGR, intrauterine growth retardation; LBW, low birth weight.

Excess salt intake is believed to be a human health risk, however, recent studies have drawn attention to the role of intrauterine environment of fetuses in growth of babies and development of diseases in adulthood. There has been an increasing number of studies suggesting that salt restriction during pregnancy has a critical influence on the intrauterine growth and development of organs of fetuses and probably switches on important factors involved in the onset of adult-type diseases through anatomical changes and hemodynamic or hormonal control including intracellular signal transduction and gene modification. These links are illustrated in Fig. 3. Salt is one of the integral components for normal growth of fetuses. Salt restriction during pregnancy is connected to IURD or death, low birth weight, organ underdevelopment and dysfunction in adulthood probably through gene-mediated mechanism. Currently, the hypothesis appears more complicated, yet, understanding the mechanisms that program fetuses that leads to adult diseases is expected to provide a new insight for pathogenesis of cardiovascular and metabolic diseases.
